# Family caregiving as a pathway to strengthen health outcomes in India

**DOI:** 10.3389/fpubh.2025.1666386

**Published:** 2025-11-06

**Authors:** Shirley Du Yan, Tanaya Jagtiani, Poornima Sharma, Arjun Rangarajan, Bhavana Issar, Madhura Kanjilal, Prachi Deo, Smriti Rana, Seema Murthy, Shahed Alam, Nachiket Mor

**Affiliations:** 1 Noora Health, San Francisco, CA, United States; 2 Noora Health India Private Limited, Bangalore, India; 3 Caregiver Saathi Foundation, Maharashtra, India; 4 Nayi Disha, Hyderabad, India; 5 Pallium India, Kerala, India; 6 Banyan Academy of Leadership in Mental Health, Chennai, India

**Keywords:** caregiving, family caregiver, caregiver support, health system strengthening, India, informal care

## Abstract

Although India’s health outcomes have improved, progress can still be made to reduce the health burden from tuberculosis, noncommunicable diseases, and maternal and neonatal health. In order to address challenges such as healthcare workforce shortages or sub-optimal time with healthcare workers, health systems can involve family caregivers who are already playing an active, informal role to support patients. Their formal involvement to support patients has been associated with improved health outcomes in health conditions. Formal support for family caregivers aligns with other priority health strategies, such as self-care, universal healthcare, and shifting demographic trends in India. Inspiration can be drawn from existing interventions that support family caregivers: training and education delivered through the public health system, caregiver support groups, community volunteers to support palliative patients, and interventions for specific patient populations (i.e., palliative care or for children with disabilities). For India, though there are no comprehensive policies that finance informal caregiver inclusion, there are examples to draw from globally. Finally, we recommend tenets of how to best engage with family caregivers, which can lead to meaningful caregiver involvement for improved health outcomes: leveraging trusted sources, focusing on actionable skills, providing just-in-time engagement, designing for diverse contexts, and ensuring caregiver safety.

## Introduction

Globally, tuberculosis, noncommunicable diseases (NCDs), and maternal and neonatal disorders are major factors in death and disability (1). Within India, NCDs and neonatal disorders are among the top 10 causes of death, with NCDs accounting for an increasing proportion of overall mortality (63% of all deaths) (2). Despite substantial improvement in health indicators such as maternal mortality, infant mortality, and child vaccination rates (3, 4), the absolute maternal mortality and infant mortality remain high (99 deaths per 100,000 live births and 26 per 1,000 live births, respectively) (5, 6). To effectively manage and reduce this disease burden, there is a need for innovative multi-stakeholder, multi-sectoral interventions at all levels of the health system.

One such strategy is the *formal* integration of family caregivers into disease management and prevention efforts. A caregiver is someone who supports a patient throughout their illness, recovery, or health event. Caregiver classifications vary by their relationship to the care recipient (spouse, child, neighbor, or professional), by care setting (home-based, community, or institutional), or by the intensity of involvement (primary, secondary, or respite) (7–9). Within India’s socio-cultural milieu, family members—especially women—play an important, albeit informal, role in providing both preventative and curative care (10, 11).

This paper argues that providing skills-based training, tools, financial compensation, and ultimately recognition to caregivers is a low-resource, high-impact way for the Indian health system to improve both short- and long-term health outcomes. Additionally, this paper does not include primary data and instead leverages Indian caregiver models to provide practical recommendations.

## Locating caregivers and their role in improving health outcomes

Despite their vital and inherent role, caregivers remain invisible within health systems, often unrecognized, unsupported, and uncompensated for the time and care they provide. This lack of support increases their stress and anxiety, as they struggle to manage tasks like managing medications and coordinating with providers while supporting patients in health facilities (12). In 18 countries, representing 50% of the world population, providers only spend an average of 5 min or less with patients—a limited time to be fully prepared to return home (13). This can lead to confusion, leaving families unsure of how to properly care for patients (14). They may miss identifying warning signs or complications that require timely care by qualified health professionals.

In India, accurately estimating the number of caregivers is difficult due to the informal nature of caregiving. One estimate puts it at 273 million people, which is approximately 20% of the population (15). This is expected to grow as the population ages and demand for healthcare increases, highlighting the enormous scale of informal caregiving in the country.

Greater family caregiver engagement can have considerable benefits for stakeholders across the healthcare system (16). Though it comes with unique challenges and barriers, family caregiver engagement can support healthcare worker shortages (16). Greater involvement of new parents in neonatal intensive care units has improved baby health outcomes (17). Many studies have explored the increasing importance of family caregivers across various types of clinical conditions spanning maternal and child health, mental health, and acute care settings, and linkages of their involvement to improved behavior change or health outcomes (18–20). Formal engagement with caregivers is increasingly important given the anticipated shortage of 10 million healthcare workers globally and 1.8 million in India by 2030 (21).

While formally integrating family caregivers into the health system offers many advantages, there are barriers and potential consequences. Determeijer et al. (22) identified seven key obstacles family caregivers face: caregiver burden, discouraging hospital environment, economic strain, ineffective collaboration with health workers, lack of support, sacrifice of personal life to provide care, and inadequate preparation for caregiving (22). Shortages of trained staff, inadequate funding, and lack of training for both caregivers and health workers impede integration efforts (23–27). Economic hardship limits caregivers’ ability to engage in health system activities (25, 28–30). Formalizing family caregivers risks increasing the burden of unpaid or underpaid labor, especially for women. The International Labour Organization’s 5R framework offers guidance to mitigate these risks: recognize, reduce, and redistribute unpaid care; reward paid care; and ensure representation (31). Complementary gender-sensitive measures can also be considered, like social security benefits, employment protections, in-kind services, and market-based incentives (32).

**Figure 1:**
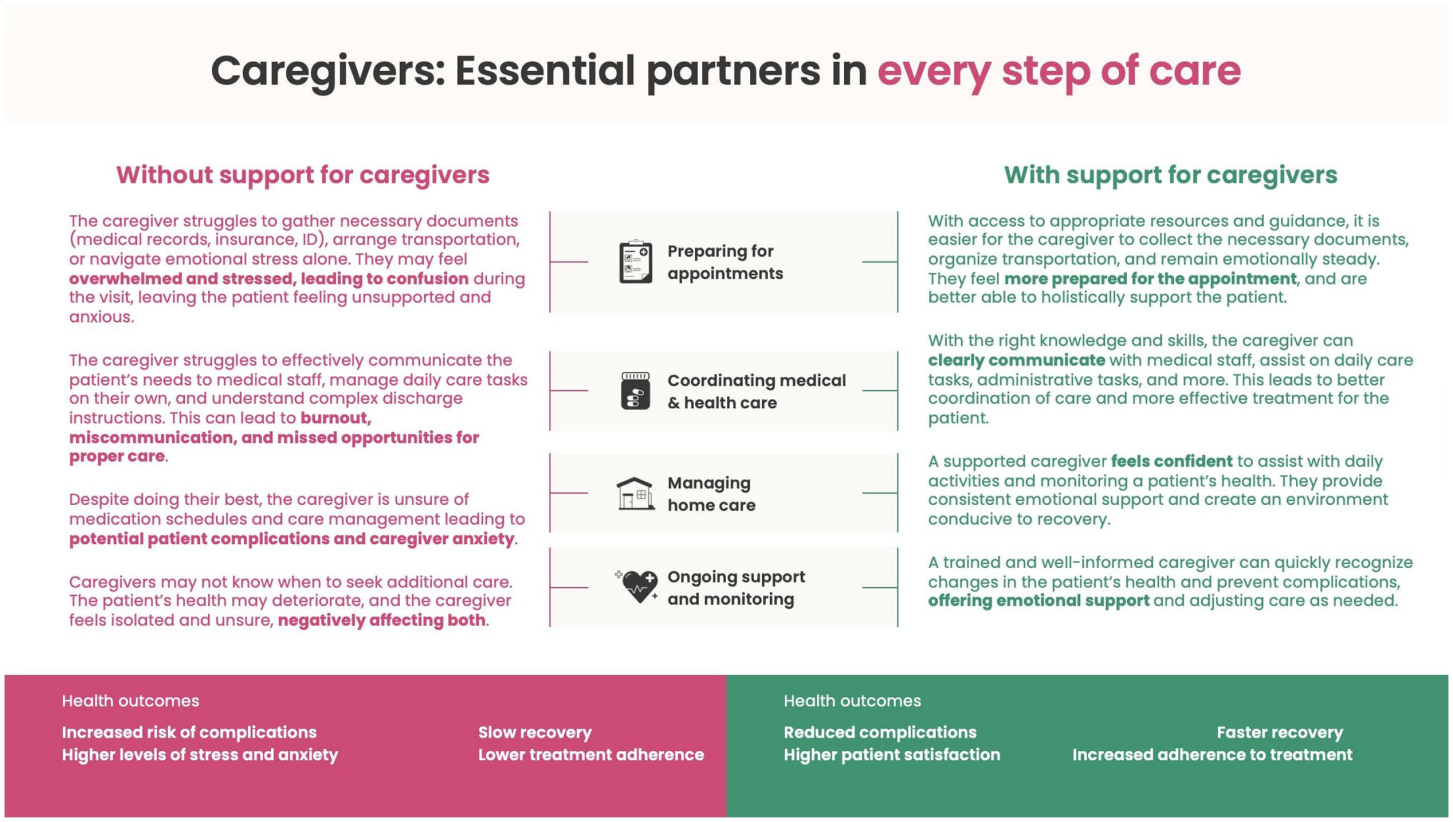
How supporting caregivers enhances their wellbeing and capabilities, ultimately improving patient health outcomes.

Importantly, such integration must also clearly define appropriate boundaries for caregiver involvement. Any activity that is complex or requires a specialized medical skill set should be done by formal health workers. Overall, coordination with healthcare workers and systems is critical for caregivers to provide a continuum of care. Figure 1 summarizes the caregivers’ critical role.

## Caregiver involvement to support the health system

Formalizing family caregivers’ roles offers a practical way to reinforce several of the World Health Organization (WHO) Health System Building Blocks (33). By recognizing caregivers as part of the health workforce—through structured training and supportive policies—health systems can expand their human resources, address workforce shortages, and create clearer pathways for collaboration with other healthcare providers. Caregiver involvement can help realize self-care, achieve primary care goals, and universal healthcare, and address health requirements from shifting demographic trends.

### Increasing self-care interventions

Complementing provider-led care, there are many health issues that can be prevented and managed by people themselves. When done with the right support, family caregiver involvement can help reduce harm and improve patient safety. Aligning with the WHO’s Patient and Safety Plan, patient and family engagement are outlined as one of the key strategies to improve patient safety (34).

### Improving primary care and universal health care

Building on the four Starfield principles, Mor and colleagues find that there are 10 sub-principles critical to delivering highly effective primary care (35). Family members could be considered most effective at (a) ensuring adherence to treatment recommendations and (b) providing guidance on the cultural ecology of health within which patients and families are situated, to the rest of the health system (36). While the latter contribution’s value may be challenging to estimate, the former contribution is sufficiently concrete that it should be possible to estimate the health system costs associated with non-adherence and to develop a mechanism through which the family member contribution to its reduction, as a substitution of the efforts of the Community Health Worker, may be estimated. The Circles of Care offer a potential pathway to integrate family caregivers at the primary care level (37). Family caregivers can support realizations for universal health coverage (UHC) and support healthcare workers.

### Preparing for shifting demographics

India’s disease burden is expected to shift. First, as India progresses from a low-income to a middle-income country, health needs shift from communicable diseases to also including non-communicable diseases. Additionally, people are expected to live longer, which is accompanied by additional health needs. Even though India has the largest youth population, the country is rapidly aging. By 2050, there will be 347 million senior people (those 60 and older), up from the current 153 million, which is especially seen in Kerala and Tamil Nadu, which have significant aging populations over 60 (38, 39). Furthermore, because older persons are likely to need healthcare services more frequently, medical costs for this age segment are more than doubled (40).

## Models for caregiver inclusion, integration, and involvement

Despite numerous challenges, a growing number of mission-driven organizations and initiatives are actively working to support caregivers. These models, implemented at different scales, demonstrate how family and community caregivers can be meaningfully integrated into health systems. Although many caregiver integration models exist, the organizations and approaches featured in this paper were selected based on the authors’ prior professional familiarity with them.

### Training caregivers in the hospital and at home

In partnership with public health systems, Noora Health’s Care Companion Program offers medically accurate, culturally appropriate skills-based training to patients and caregivers, enabling them to manage health conditions effectively at home. Furthermore, families can sign up for a mobile-based service, which continues to offer crucial information once they have left the hospital and provides a platform for them to ask questions and receive guidance tailored to their specific needs across the continuum of care and health conditions.

Over the last 10 years, the Care Companion Program has trained more than 33 million caregivers and patients across over 11,700 healthcare facilities in nine Indian states. It has demonstrated significant improvements in patient outcomes, such as a 71% reduction in post-surgical cardiac complications and a 56% decrease in newborn readmissions (41, 42).

Take the story of a caregiver from Nashik, Maharashtra, who supported her uncle, a patient at Nashik Civil Hospital, for high blood pressure and diabetes. During a visit to the hospital, she attended a Care Companion Program session in which a nurse was explaining how to recognize and monitor the telltale signs of diabetes and hypertension. She recognized she had experienced these symptoms herself, immediately sought medical help, and learnt to take care of her own health in addition to caring for others. After confirming her diagnosis with doctors for both hypertension and diabetes, she changed her lifestyle and proactively started managing her health. Today, with support from their mobile-based service, she not only manages her own health but has also become an advocate in her community.

### Enhancing practical home-based care

In partnership with the National Health Systems Resource Center, Pallium India developed a comprehensive training program to upskill frontline health workers on supporting patients requiring care at home, such as those with advanced or chronic illnesses. The training modules, complemented by over 60 instructional videos, cover everything from oral care for conscious and unconscious patients to prevention of bed sores, and much more (43). Equipped with these tools and the right knowledge, caregivers are able to better manage their tasks, reduce the anxiety associated with them, and provide much-needed non-pharmacological support to patients. In 2024, they reached approximately 5,000 patients via their clinical program and 2,711 patients via their telehealth service.

Additionally, for people living with terminal or chronic illnesses, the program helps families build advance care plans to prepare for any potential emergencies that may occur at home, such as a loss of consciousness or a drop in blood pressure. These plans are customized to the illness and align with the family’s preferences and values, reducing the emotional burden on caregivers and making decisions easier during a critical time.

### Strengthening care through community support and policy integration

Kerala’s community participation palliative care model demonstrates how governments can support home-based care and positively impact the caregiver burden. Through this approach, community volunteers are provided with training to recognize palliative care needs patients may have, such as procurement of medicines and household items, or providing logistical support, transporting patients to health facilities. During the COVID-19 pandemic, volunteers included pathologists and diagnostic clinic staff who would help with basic tasks like collecting blood samples to monitor certain health parameters. Volunteers also play a major role in mitigating isolation and loneliness. For older caregivers, a comprehensive geriatric assessment helps in monitoring mobility and other parameters to prevent health-related problems that could lead to further caregiving complications. Each of these interventions is bolstered with additional support by way of telehealth helplines staffed by interdisciplinary teams of physicians, nurses, and medical social workers.

### Building a strong caregiver support network

Caregiver Saathi seeks to alleviate the caregiving burden by helping family members decouple their role from their identity; seek the necessary support or acquire skills through educational content, training programs, practical tools; and access supportive community and professional home-care services to help caregivers lead fuller, healthier lives (43). They collaborate with workplaces, healthcare institutions, professional caregivers, healthcare practitioners, care-service providers, employers, and well-wishers to build a robust network of care and enable effective caregiving practices. Through these efforts, caregivers feel seen, supported, and empowered to provide compassionate care. Having reached over 54,000 caregivers, Caregiver Saathi focuses on advocacy and awareness; wellbeing and healing; education and guidance; community building; professional services; and storytelling.

### Supporting families of people with disabilities

As estimated 31 million individuals in India have intellectual and developmental disabilities such as autism or Down syndrome (45). Providing them with care and support is a lifelong journey for family caregivers. However, social stigma, a lack of awareness, and limited services make it harder for family caregivers to provide the support their loved ones require. Nayi Disha bridges this gap by providing families with accurate information, expert guidance, and a supportive community to help them navigate their caregiving journey.

Take the example of a mother from Raipur, Chhattisgarh, whose three-year-old son was diagnosed with autism. After attending a rights-based awareness session hosted by Nayi Disha, she learned about her child’s legal rights and the government schemes available to support him. She also began using their website resources to better understand her son’s condition. Alongside, she attended Saksham, a multi-day parent training program where she learnt skills for providing effective care for her son at home. Furthermore, she was connected with hundreds of other families through WhatsApp peer support groups. Together, these interventions allowed her to support her child’s development and her own wellbeing holistically. Access to reliable resources and a strong community helped her transform uncertainty into confidence and isolation into shared strength. Nayi Disha has reached 9,00,000 people through its digital platform, alongside the 62,000 families it actively supports in accessing various services.

Table 1 summarizes different parameters for each of these caregiver engagement models, and Supplementary Figure A1 outlines a summative journey informed by collective model experiences.

**Table 1:** Comparative overview of select caregiver engagement models in India.

Model/program	Scale and reach	Key outcomes	Sustainability challenges	Strengths	Limitations	Transferability to other LMICs
Noora Health —Care Companion Program	33M+ caregivers trained across 11,700 facilities in 9 states	71% reduction in post-surgical cardiac complications; 16% decrease in newborn complications (41, 42)	Relies on the public health facility and system buy-in, sustaining digital engagement	Evidence-based, scalable, measurable health outcomes	Needs integration with public health facility and system; requires digital follow-up	High—adaptable to other LMIC health systems
Pallium India—Home-based Palliative Training	5,000 patients via clinical program; 2,711 via telehealth (2024)	Reduced caregiver anxiety; better non-pharmacological support for patients (43)	Funding constraints; specialist training needs	Builds frontline skills; holistic, person-centered approach	Resource-intensive; small scale relative to need	Moderate—feasible where community health workers can be trained
Kerala—Community Palliative Care Model	Statewide volunteer-driven network	Reduced caregiver isolation; improved continuity of home-based care (43, 49)	Volunteer burnout: dependence on community cohesion	Strong state support; mobilization of community volunteers	Relies heavily on social capital and volunteerism	Moderate—transferable where strong civic networks exist
Caregiver Saathi	54,000 caregivers supported; advocacy and awareness reach wider	Improved caregiver wellbeing, advocacy gains, stronger community ties (44)	Fragmented ecosystem; limited integration with formal policy	Provides emotional and identity support; cross-sector collaborations	Scale still limited; not clinically embedded	High—flexible for workplaces and advocacy networks
Nayi Disha	9,00,000 reached digitally; 62,000 families supported in person	Increased parental skills; reduced stigma; improved access to disability schemes (45)	Digital divide: sustaining peer networks	Rights-based, digital-first, peer support groups	Access barriers for rural/low-literacy families	High—adaptable with localization and translation

## Financing caregiver inclusion

Mor and Shukla estimate INR 2000 per capita to deliver UHC commitments, for which 12 states spend more than that, and by 2030 should be possible (46). Ayushman Bharat, State Level Insurance, and National Health Mission are all smaller components of the overall government health expenditure - the most significant components are the resources allocated by state governments from their own budgets. Based on PRS India budget analysis, health allocations are ~6% of annual budgets, not decreasing to pre-COVID-19 levels (see Supplementary Figure A2) (47). This suggests India has enough funds to compensate family caregivers for their critical care within the healthcare system. For example, within India, Kerala has a provision for subsidizing electricity bills for households that have patients dependent on electricity-driven devices like oxygen concentrators and airbeds (48, 49). Additionally, in 2024, Karnataka provides INR 1,000 per month for caregivers of selected illnesses and diseases (50).

Globally, there are different examples of how to finance caregiving-related costs (51). In Canada, it is estimated that replacing formal services with family caregiver contributions represents 5% of its GDP, or a third of its government healthcare expenditure (52). While many long-standing consumer-directed personal assistance programs in the U. S. exclude supporting family caregivers explicitly exclude “legally responsible individuals” (such as parents and spouses) from the policy’s remit, but permit them to hire other certified people who can be paid by the government at twice the minimum wage (53).

In resource-constrained countries like India, where high inequalities create welfare demands that far exceed government capacity, welfare-oriented approaches, like the ones outlined above, are likely to be less compelling (54). The best strategy would be to link it back to health system outcomes and to have the payment to family caregivers come out of the health budgets using what Fast and colleagues describe as a “replacement cost method” in which the value of family care work is the price of time spent on care (52). Several other countries, like the United States and Uruguay, have programs to reimburse caregivers for their time (55–58).

## Key tenets of working with caregivers

The following key tenets for working with caregivers are proposed:

- Leverage trusted sources: Healthcare providers, from community healthcare workers to hospital nurses, can be trained to impart key health skills to family caregivers—who are often more receptive to health behavior change when receiving information from a trusted health professional.
- Focus on actionable skills: Health information should be tailored to encourage behavior change and the practical application of caregiving skills, ultimately targeting improved health outcomes. Frame caregiver engagement programs as training (as opposed to education) and skill building (as opposed to information sharing), for a meaningful focus is placed on tangible actions that caregivers can practice.
- Provide just-in-time engagement: Patients and families seek care in a variety of ways and settings. Behavior adoption is greater when the skills and training are provided when families need it most. This could mean within the context of healthcare delivery or services, or within one’s own community setting at the time of a health need.
- Design for diverse contexts: Understanding and working with existing resources, settings, and cultural norms is essential in designing more meaningful solutions for family caregivers. While evidence-based practices can be standardized across populations, programs must be adjusted to these cultural considerations.
- Ensure caregiver safety: Caregiving is difficult work, and families are at increased risk for physical and mental health consequences of their care tasks (59). There is a balance that must be struck, where and new family caregivers may be equipped to take on healthcare responsibilities while at the same time not adding additional burden to their own health and wellbeing.

## Conclusion

The vital role that family caregivers play in managing illness and supporting recovery cannot be overstated. With India’s high disease burden, the public healthcare system faces overwhelming challenges. Yet, caregivers—often unrecognized and unsupported—continue to bear the immense responsibility of providing life-saving care, often at great personal cost. This paper has shown that addressing the gaps in caregiver support—whether financial, emotional, or skills-based—is not only crucial for patient wellbeing but also for supporting healthcare workers and the overall health systems sustainability. Low-resource and high-impact models for caregiver engagement already exist and have proven effective. Expansion of these initiatives can ensure caregivers receive the recognition, resources, and compensation they deserve. Additional research to demonstrate the impact of caregiver involvement on health system improvement, caregiver burden, and cost-effectiveness analyses, patient outcomes and long-term implications for health system efficiency can enrich the understanding and value of caregiver involvement. This is a call to action for policymakers, healthcare professionals, and society at large: It is time to invest in caregivers, not just as family members but as equal partners in healthcare. The wellbeing of millions depends on it.

## Data Availability

The original contributions presented in the study are included in the article/supplementary material, further inquiries can be directed to the corresponding authors.
