# Correction: Family caregiving as a pathway to strengthen health outcomes in India

**DOI:** 10.3389/fpubh.2025.1765128

**Published:** 2026-01-09

**Authors:** Shirley Du Yan, Tanaya Jagtiani, Poornima Sharma, Arjun Rangarajan, Bhavana Issar, Madhura Kanjilal, Prachi Deo, Smriti Rana, Seema Murthy, Shahed Alam, Nachiket Mor

**Affiliations:** 1 Noora Health, San Francisco, CA, United States; 2 Noora Health India Private Limited, Bangalore, India; 3 Caregiver Saathi Foundation, Maharashtra, India; 4 Nayi Disha, Hyderabad, India; 5 Pallium India, Kerala, India; 6 Banyan Academy of Leadership in Mental Health, Chennai, India

**Keywords:** caregiving, family caregiver, caregiver support, health system strengthening, India, informal care

In the Acknowledgements, the names of two people “Charley Vincent” and “Simran Toor” were missed.

It originally stated:

“We would like to thank Rohina Thapar, Ramachandran K M, and Manju Pothen for their support with visuals in this manuscript. We also thank Keri Wachter and Jim Determeijer for their review of this manuscript.”

The correct statement is:

“We would like to thank Rohina Thapar, Ramachandran K M, Manju Pothen, Charley Vincent, and Simran Toor for their support with visuals and research insights in this manuscript. We also thank Keri Wachter and Jim Determeijer for their review of this manuscript.”

The original version of this article has been updated.

