# Supplementary material for: Family caregiving as a pathway to strengthen health outcomes in India
Source: Front Public Health. 2025 Nov 6;13:1666386. doi: 10.3389/fpubh.2025.1666386 (PMC12631336; doi:10.3389/fpubh.2025.1666386)
Supplement: Supplementary file 1 [file Supplementary_file_1.pdf]

Appendix

Figure 1: An illustrative example of a caregiver journey

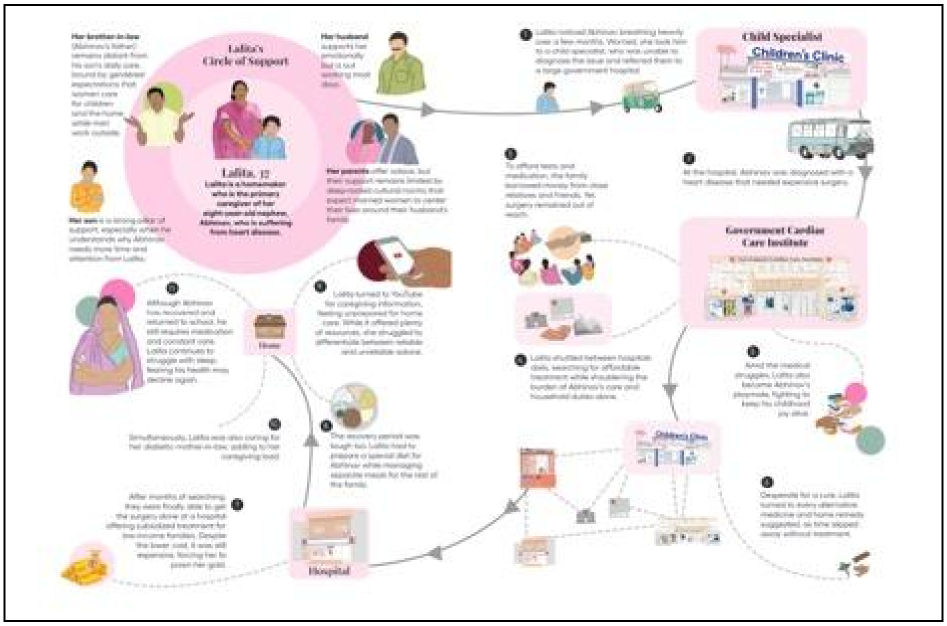

This visual narrates the caregiving journey of Lalita, a 37-year-old homemaker and primary caregiver to her 8-year-old nephew, Abhinav, who has been diagnosed with a serious heart condition. It captures not only the challenges and critical decisions Lalita faces, but also the limited and uneven support she receives as she navigates a complex and emotionally demanding caregiving experience.

Figure 2:

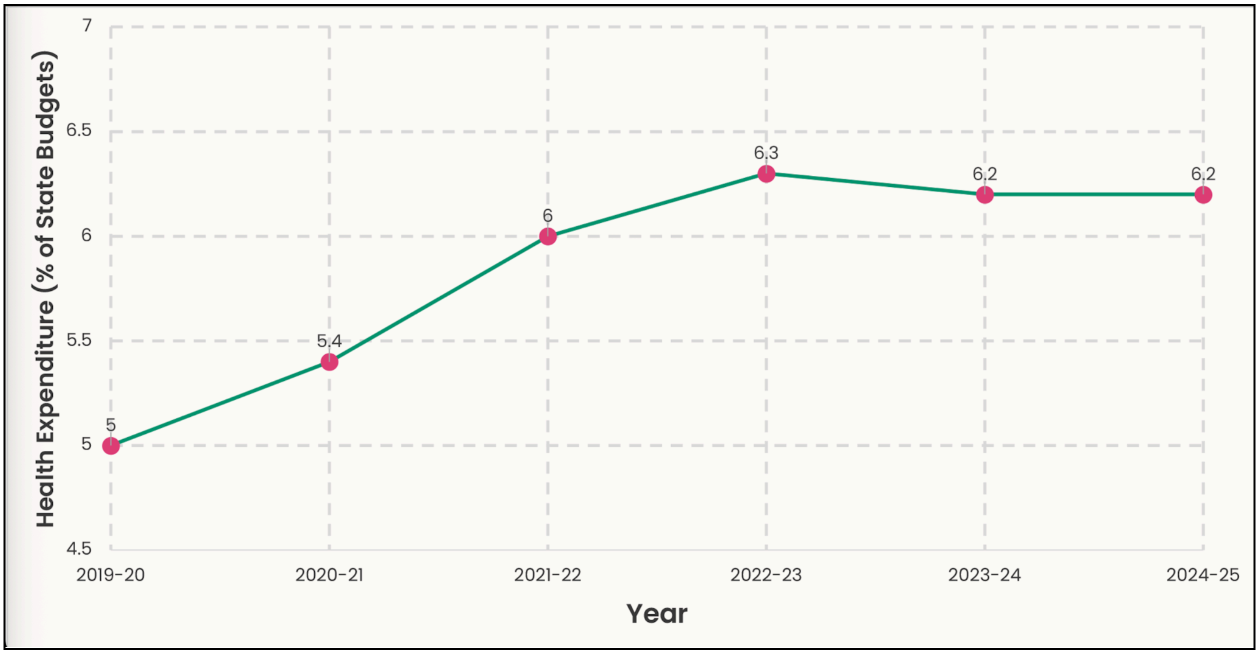

This graph shows the average share of state budgets allocated to health from 2019–20 to 2024–25.
